# Post-surgical Hearing Improvement in a Case of Jugular Foramen Schwannoma Despite the Lack of Response in a Preoperative Auditory Brainstem Response Test

**DOI:** 10.7759/cureus.61623

**Published:** 2024-06-03

**Authors:** Yudai Morisaki, Fumihiko Nishimura, Ryosuke Matsuda, Tsunenori Takatani, Ichiro Nakagawa

**Affiliations:** 1 Neurosurgery, Nara Medical University, Kashihara, JPN; 2 Anesthesiology, Nara Medical University, Kashihara, JPN

**Keywords:** otorhinolaryngology, neurosurgery, hearing improvement, auditory brain response, jugular foramen schwannoma

## Abstract

We report a rare case involving improved hearing after surgery for a jugular foramen schwannoma despite the lack of response during the preoperative auditory brainstem response (ABR) test. A left jugular foramen tumor was diagnosed in a 79-year-old man with hearing loss. No response was observed during the preoperative ABR test. However, his hearing improved after surgery using the lateral suboccipital approach. Following Gamma Knife radiation to the residual tumor post-surgery, the ABR test detected V waves. The hearing of patients with cerebellopontine angle tumors can improve even when there is no response during the preoperative ABR test.

## Introduction

Intracranial schwannomas account for 8% of primary intracranial tumors; 94.5% of these schwannomas are acoustic neuromas and 2.9% are jugular foramen schwannomas [1-3]. Evidently, jugular foramen schwannomas are rare entities. Jugular foramen schwannomas often present with hearing loss; however, this can improve after surgical treatment [4-6]. The auditory brainstem response (ABR) test is highly reproducible and often used clinically to objectively evaluate hearing and diagnose impaired areas. Most cases of hearing loss associated with jugular foramen schwannomas involve compression and stretching of the cochlear nerve in the cisternal region, which results in posterior labyrinthine hearing loss. Therefore, during the ABR test, the I wave often remains normal, suggesting a posterior labyrinthine disorder. In some cases involving inner ear damage, the ABR test shows the absence of I waves, indicating a poor prognosis for hearing [5]. We report a case of a jugular foramen schwannoma with V waves and effective hearing improvement in the postoperative ABR test despite a lack of response in the preoperative ABR test.

## Case presentation

The patient was a 79-year-old male with a history of right cholesteatoma surgery and hearing loss in his right ear for 10 years. He was living with a hearing aid in his right ear. One year prior, he had become aware of hearing loss in his left ear which gradually worsened, leading him to visit a local doctor. He had been diagnosed with a brain tumor and referred to our department. He did not have neurofibromatosis type 2 (NF2). He was conscious, had no hearing in his right ear, and could only hear in his left ear with a hearing aid. He reported very mild dysphagia and light-headedness.

Preoperative examination

**Figure 1:**
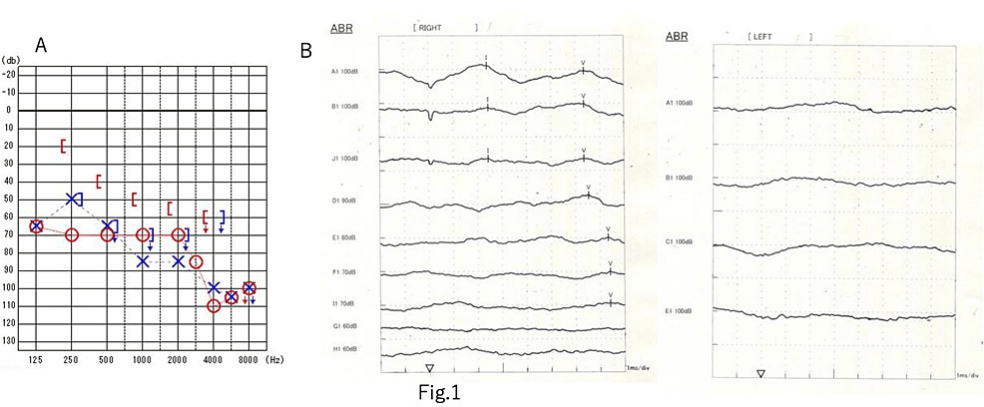
Preoperative examination A: preop pure tone audiometry. According to the 4-division method, the right side was 70 dBnHL, the left was 80 dBnHL, and the hearing loss was worse than high-pitched sounds on both sides. B: preop auditory brainstem response (ABR). The right I wave latency was 2.42 ms, the V wave latency was 6.36 ms, the I-V wave latency was 3.94 ms, and the left side showed no response

**Figure 2:**
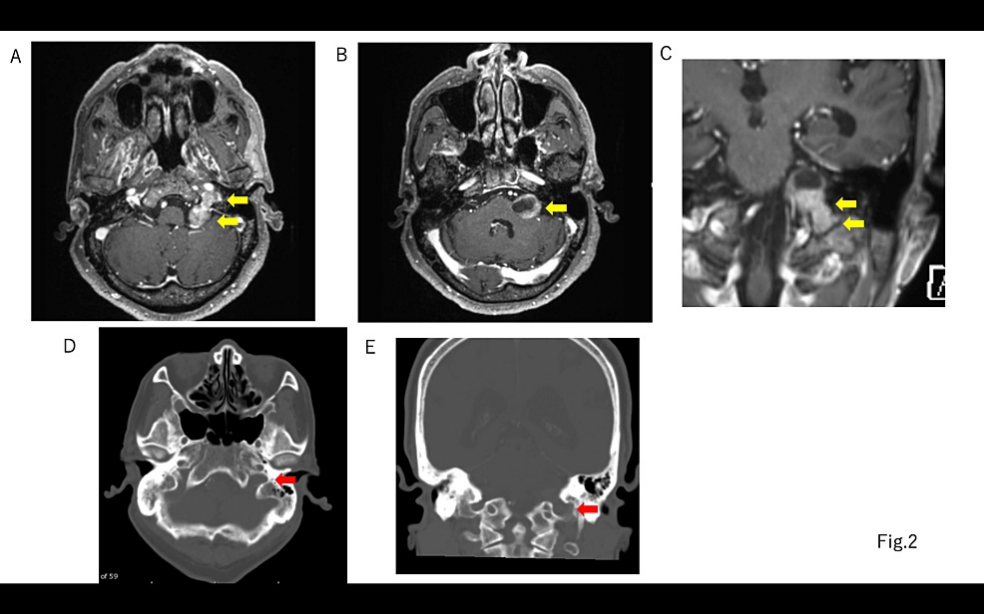
Preoperative gadolinium (Gd) MRI and CT bone images A, B: the tumor was an intracranial and extracranial dumbbell type with an inner cyst. C: Gd MRI coronal image. D: axial plane CT bone image. E: coronal plane CT bone image. Yellow block arrow: jugular foramen tumor. Red block arrow: enlarged jugular foramen CT: computed tomography; MRI: magnetic resonance imaging

The hearing test revealed 70 dBnHL on the right side and 80 dBnHL on the left side using a 4-section system (Figure 1A). ABR had a right I wave latency of 2.42 ms, a V wave latency of 6.36 ms, an I-V wave latency of 3.94 ms, and the left side was unresponsive (Figure 1B). A head-enhanced MRI revealed a relatively uniformly enhanced dumbbell-shaped lesion measuring 25 x 22 mm in the left cerebellopontine angle extending into the jugular foramen, with an internal cyst (Figure 2). The tumor did not extend into the internal auditory canal. Based on contrast-enhanced cisternal images, the cranial nerves (CNs) VII and VIII were suspected to be tumors running cranially and dorsally.

Operation

**Figure 3:**
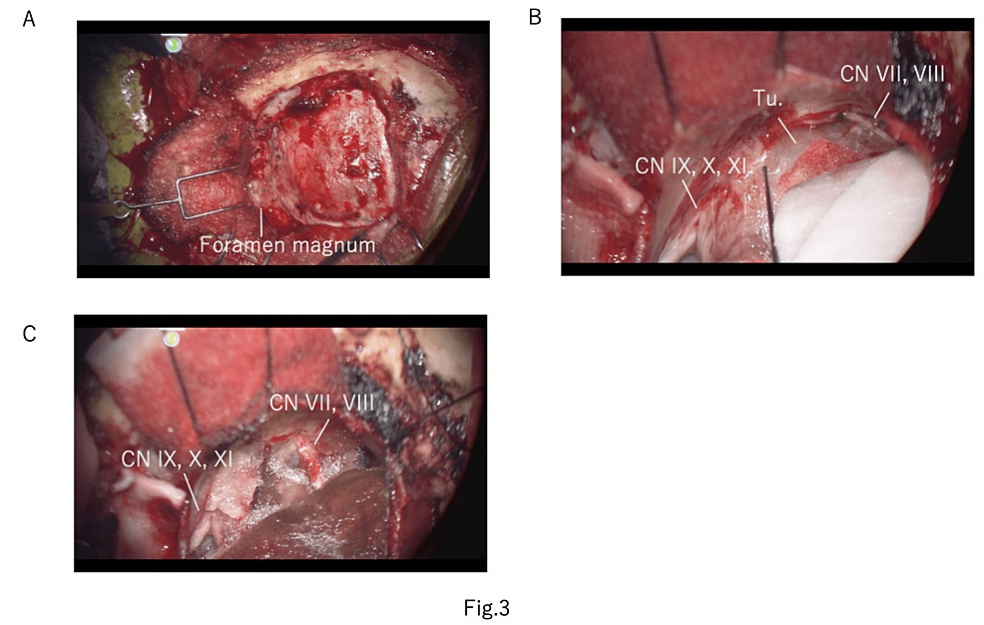
Intraoperative images A: left lateral suboccipital craniotomy with foramen magnum opening. B: image before tumor resection. C: final image after tumor removal CN: cranial nerve; Tu.: tumor

The surgery was performed adopting a lateral suboccipital approach (Figure 3A). Monitoring was performed using transcranial facial motor evoked potential (Tc-fMEP) for CN VII, and intermittent direct stimulation was performed on the orbicularis oculi, orbicularis oris, and mentalis muscles. ABR was conducted for CN VIII function. Intermittent direct stimulation and continuous stimulation monitoring were performed in CNs IX, X, and XI. Using intubation tubes, needle electrodes were placed in the pharyngeal, tongue, and vocal cord muscles. ABR on the left side was unresponsive as in the preoperative examination. When intermittent direct stimulation was performed, a response of the vocal cord muscles was observed on the caudal side of the tumor, confirming an intact CN X. Additionally, CNs VII and VIII were confirmed cranially - dorsal to the tumor (Figure 3B). Under continuous monitoring of CN X, intra-tumoral decompression was performed between CNs X and VIII. Upon intermittent direct stimulation, responses were observed in the cranial part of CN VII and the caudal part of CN X within the tumor, at 0.5 mA each. As the monitoring findings of CNs VII and X were somewhat concerning, we decided to perform a subtotal resection of the tumor given the risk of nerve damage (Figure 3C). The tumor within the jugular foramen was not removed. During the surgery, the left ABR remained unresponsive throughout, and no other deterioration in monitoring was observed. The tumor was found to originate from the sheath of the left glossopharyngeal nerve under careful microscopic inspection. 

Postoperative course

**Figure 4:**
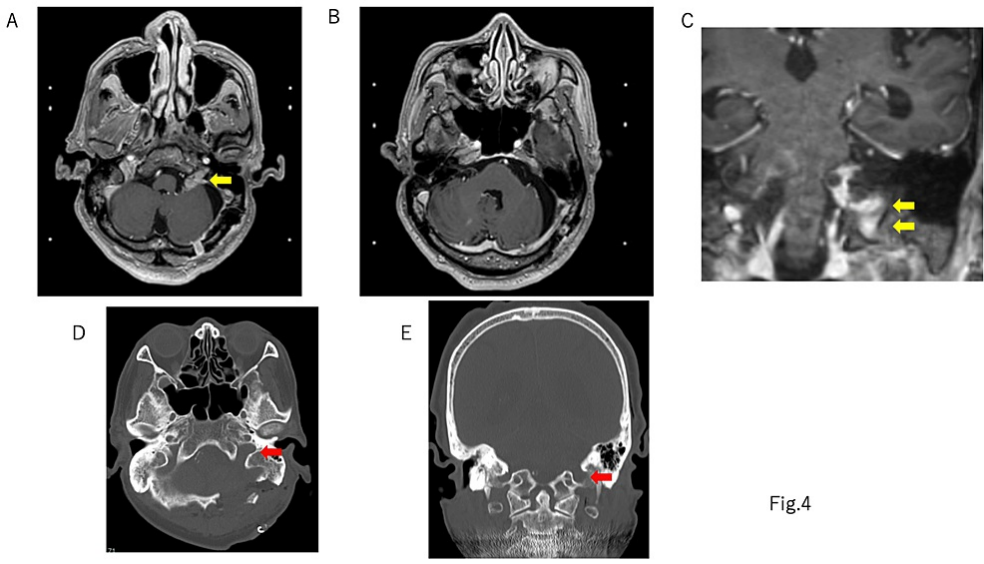
Postoperative gadolinium (Gd) MRI 80% of the cisternal portion of the tumor was removed, but the jugular foramen portion of the tumor was left. A, B: axial plane Gd MRI. C: coronal plane Gd MRI. D: axial plane CT bone image. E: coronal plane CT bone image. Yellow block arrow: residual tumor. Red block arrow: enlarged jugular foramen MRI: magnetic resonance imaging

**Figure 5:**
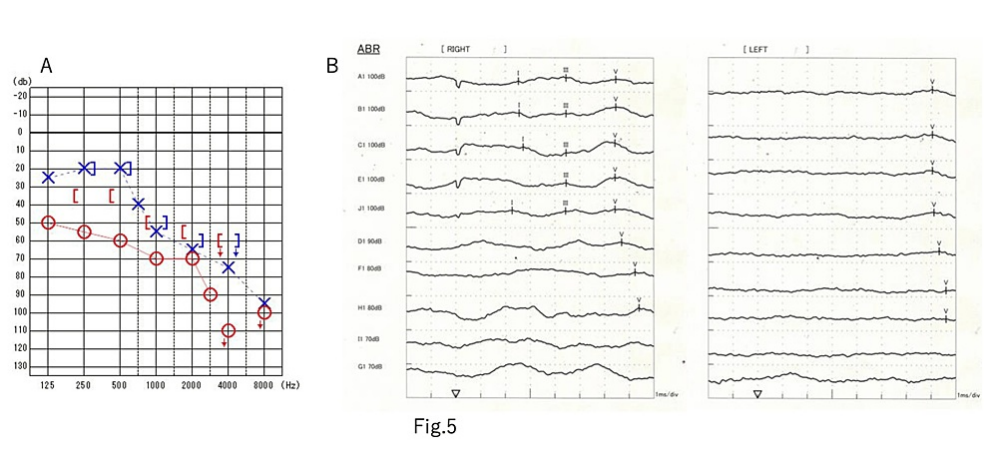
Postoperative audiological examination A: postop pure tone audiometry. The 4-division method revealed an improvement in the left side, with 67.5 dBnHL on the right and 48.8 dBnHL on the left. There was an improvement overall, but especially in the low-pitched sounds. B: postop ABR test. A V wave appeared and the latency was 7.60 ms ABR: auditory brainstem response

Soon after surgery, the patient’s left hearing improved and he was able to hear without hearing aids. No facial nerve paralysis or dysphagia were observed, and the patient's light-headedness improved. Postoperative contrast-enhanced MRI revealed that 80% of the tumor had been removed, with some portion remaining in the caudal side of CNs VII and VIII and near the lower CNs (Figure 4). No postoperative cerebrospinal fluid leak and hydrocephalus were observed. The patient was discharged home, where he lived on his own, and the residual tumor was treated using Gamma Knife radiation (12Gy) 1.5 months after the surgery. Postoperative audiometry showed an improvement in hearing on the left side, with 67.5 dBnHL on the right and 48.8 dBnHL on the left (Figure 5A). A V wave appeared on the ABR test, and the latency was 7.60 ms (Figure 5B).

## Discussion

Significance of ABR

We reported a case of a male patient with a jugular foramen tumor who had no effective hearing preoperatively and whose preoperative ABR test was unresponsive; however, his hearing improved postoperatively. ABR is considered to be an excellent test for objectively evaluating hearing ability and diagnosing the location responsible for hearing loss before surgery. Several factors may lead to inner ear disorders. Common syndromes that may cause hearing loss include Usher, Jervell, Lange Nielsen, CHARGE, Waardenburg, Pendred, Goldenhar, and DiGeorge. Ototoxicity of the drug gentamicin may also cause inner ear damage. Also, isolated malformations of the inner ear, such as the absence of the cochlear septum (Mondini syndrome), a single vestibular cavity (Michel syndrome), and hypertrophy of the vestibular aqueduct, cause cochleovestibular lesions. Congenital cytomegalovirus infection may impair cochlear function [7]. The I wave is generated by the cochlear auditory nerve; its disappearance indicates a disorder in the inner ear.

The loss of hearing in vestibular schwannomas and cerebellopontine angle tumors is thought to constitute a combination of inner ear and cochlear nerve disorders [6,8]. Inner ear disorders are thought to result from sensory epithelium damage and biochemical changes in the inner ear lymph fluid due to inner ear artery occlusion or ischemia caused by blood vessel compression in the inner auditory canal. The main cause of cochlear neuropathy is thought to be conduction block, caused by cochlear nerve compression by tumors [9]. In many cases where hearing recovers after surgery, the preoperative cochlear function is good, and the preoperative hearing loss is attributed to conduction block secondary to cochlear nerve compression caused by the tumor. In our case, preoperative inner ear function tests such as distortion product otoacoustic emissions (DPOAEs) were not performed; therefore, preoperative inner ear function was unknown. However, since the patient’s hearing recovered after the tumor was removed, it is thought that the cause was not inner ear ischemia or irreversible inner ear damage, but rather a conduction block in the intracisternal cochlear nerve secondary to compression.

There are two possible reasons for the ABR non-response in our case. First, previous studies have reported non-responsiveness due to poor response synchronization because of a cochlear nerve conduction block [10]. Second, ABR is known to have a decreased response in cases of hearing loss at high frequencies, as observed in our patient. These reasons may explain the ABR non-response. Although this patient had no effective hearing before surgery, he had hearing loss, especially at high frequencies, which may have affected the ABR response. In our case, hearing improved mainly in the low-frequency range, although there was also a slight improvement in the high-frequency range. The improvement in ABR after surgery may have resulted from improved hearing in the high-frequency range.

Procedures aimed at preserving each cranial nerve

We were able to preserve and improve the function of CNs VII to XI by making full use of various CN monitoring techniques. Normally, it is extremely difficult to remove a jugular foramen tumor while preserving lower CN function. Additionally, in the elderly, CN function is especially vulnerable, and intraoperative monitoring is likely to show deterioration; additionally, once swallowing function deteriorates, it directly leads to a decline in the quality of life [1]. However, preoperative symptoms of CNs caused by tumor compression may improve, especially hearing loss, dizziness, and swallowing; therefore, surgery is indicated [6]. Accurate intraoperative monitoring of the CNs is essential to overcome the conflicting propositions of worsening and improving CN symptoms due to this surgery. Matsushima et al. have reported the usefulness of continuous monitoring of lower CNs for jugular foramen tumors [11]. In our case, we were able to preserve these nerve functions by combining continuous monitoring of the lower CNs with intermittent direct stimulation monitoring of the facial nerve.

Usefulness of preoperative testing

In our case, there was no effective hearing on the affected side before surgery, and there was no response to the preoperative ABR test; thus, we thought it was impossible to preserve hearing. However, as seen in this case, there is a possibility that hearing will improve even if there is no response in the preoperative ABR test, especially in cases of cerebellopontine angle tumors without internal auditory canal extension. In particular, for patients who have hearing loss on the unaffected side, as in our patient, improvement in hearing on the affected side has a significant impact on postoperative improvement in activities of daily living. In general, it has been reported that hearing can be preserved or improved in cerebellopontine angle tumors with an ABR I waveform [2]. However, as in our case, even if there was no preoperative ABR response, hearing improves after surgery in some cases. The tumor is compressing the brain stem and cochlear nerve, and hence stereotactic radiation therapy is not expected to improve hearing. Thus, it is considered dangerous to rely solely on ABR results when planning interventions to preserve hearing. A limitation of this study is that preoperative inner ear function testing, which might have benefited the patient, was not performed. This test might have determined that inner ear function was reversible before surgery.

## Conclusions

We reported a case of a patient with a jugular foramen schwannoma in which V waves appeared postoperatively and hearing recovered despite a lack of response in the preoperative ABR test. Stereotactic radiotherapy for tumors compressing the cochlear nerve or brainstem cannot be expected to improve hearing, and surgical decompression is crucial in such cases. Postoperative CN function preservation and improvement can be achieved through accurate intraoperative neurological monitoring and surgical techniques.
